# Western Honduras Copán Population–Based Cancer Registry: Initial Estimates and a Model for Rural Central America

**DOI:** 10.1200/GO.21.00273

**Published:** 2021-12-16

**Authors:** Dalton Argean Norwood, Eleazar Enrique Montalvan-Sanchez, Juan E. Corral, Dagoberto Estévez-Ordoñez, Andrea A. Paredes, Lucia B. Domínguez, Aida A. Rodríguez, Luis E. Bravo, Douglas R. Morgan, Ricardo L. Domínguez

**Affiliations:** ^1^ Western Honduras Gastric Cancer Prevention Initiative, Hospital de Occidente, Santa Rosa de Copán, Honduras; ^2^ Universidad Nacional Autónoma de Honduras, School of Medicine, Honduras; ^3^ School of Medicine, The University of Alabama at Birmingham, Birmingham, AL; ^4^ Indiana University, Department of Medicine, Indianapolis, IN; ^5^ Division of Gatroenterology and Hepatology, Presbyterian Healthcare Services, Albuquerque, New Mexico; ^6^ IACR Regional Representative for Latin America, International Agency for Research on Cancer, Lyon, France; ^7^ Departamento de Patología, Universidad del Valle, Cali, Colombia; ^8^ Division of Gastroenterology, Hepatology and Nutrition, The University of Alabama at Birmingham, Birmingham, AL

## Abstract

**PURPOSE:**

Population-based cancer registries (PBCRs) are critical for national cancer control planning, yet few low- and middle-income countries (LMICs) have quality PBCRs. The Central America Four region represents the principal LMIC region in the Western hemisphere. We describe the establishment of a PBCR in rural Western Honduras with first estimates for the 2013-2017 period.

**METHODS:**

The Western Honduras PBCR was established through a collaboration of academic institutions and the Honduras Ministry of Health for collection of incident cancer data from public and private health services. Data were recorded using the Research Electronic Data Capture (REDCap) web-based platform with data monitoring and quality checks. Crude and age-standardized rates (ASRs) were calculated at the regional level, following WHO methodology.

**RESULTS:**

The web-based platform for data collection, available ancillary data services (eg, endoscopy), and technical support from international centers (United States and Colombia) were instrumental for quality control. Crude cancer incidence rates were 112.2, 69.8, and 154.6 per 100,000 habitants overall, males, and females, respectively (excluding nonmelanoma skin cancer). The adjusted ASRs were 84.2, 49.6, and 118.9 per 100,000 overall habitants, males, and females, respectively. The most common sites among men were stomach (ASR 26.0, 52.4%), colorectal (ASR 5.11, 10.15%), and prostate (ASR 2.7, 5.4%). The most common sites in women were cervix (ASR 34.2, 36.7%), breast (ASR 11.2, 12.3%), and stomach (ASR 10.8, 11.7%).

**CONCLUSION:**

The Copán-PBCR represents a successful model to develop cancer monitoring in rural LMICs. Innovations included the use of the REDCap platform and leverage of Health Ministry resources. This provides the first PBCR data for Honduras and the Central America Four and confirms that infection-driven cancers, such as gastric and cervical, should be priority targets for cancer control initiatives.

## INTRODUCTION

Population-Based Cancer Registries (PBCRs) are critical for cancer prevention and National Cancer Control Programs.^1,2^ They delineate the cancer burden and measure the efficacy of prevention interventions over time,^1,3^ and data quality and quality control measures are imperative.^4-8^ Despite efforts during the last two World Health Assemblies, and the action plan by the WHO in 2013,^9^ the majority of low-middle income countries (LMICs) lack quality PBCRs.^10^

**CONTEXT**

**Key Objective**
Population-based cancer registries are lacking in the Central America Four region, the principal low- and middle-income countries region in the Western hemisphere. The Copán population–based cancer registries in rural western Honduras are established, with first estimates for the 2013-2017 period.
**Knowledge Generated**
Gastric and cervical cancers dominate for males and females, respectively, followed by breast, colorectal, and prostate.
**Relevance**
The infection-associated cancers, now with the emerging cancers, portend the future double cancer burden common to the transitioning economies in Latin America, with implications for Central America Four regional cancer control planning and US immigrant populations.

In 2014, the WHO estimated that noncommunicable diseases became the leading cause of death worldwide, with cancer being a major component and the majority of the burden in LMICs.^11^ According to the International Agency for Research on Cancer (IARC), cancer burden rose to 18.1 million incident cases and 9.6 million deaths in 2018 and will become the leading cause of death in nearly every country this century.^12,13^

The Central America Four (CA-4) region, comprising Guatemala, Honduras, El Salvador, and Nicaragua, is the principal LMIC region in the Western hemisphere, with a growing noncommunicable cancer burden, increasing by 73% by 2030.^14^ Piñeros and Morgan et al summarized the nascent PBCRs in the CA-4 following the 2014 Central America Cancer Bioinformatics symposium.^14^ Efforts for cancer surveillance in the CA-4 have faced various obstacles.^14^ PBCR initiatives by the Guatemala Ministry of Health in 1995-1997 and 2013-2015 were left unfunded.^15^ Academic and nonprofit organizations have initiatives related to prevalent cancer types (eg, stomach, cervix, and breast),^16-18^ which may benefit from screening strategies in the local setting.^19,20^ Death certificates have wide variations in quality and completeness. Hospital-based cancer registries generate data for the less prevalent cancer types to serve as a bridge to the establishment of a PBCR, but are limited by variations in health care access, under-representation of rural communities, and lack of delineation of the population at risk.^14,21^ Thus, most of the IARC GLOBOCAN estimates for the CA-4 region have been imputed from neighboring countries.^22^

Honduras is rapidly growing with the third highest fertility rate in the Americas (2.46 births per woman). The initial effort to establish a PBCR in Honduras in 2005 was short-lived because of funding and shortage of trained personnel, similar to Guatemala.^23,24^ In 2002, a registry of gastric cancer cases in Western Honduras launched through academic collaboration, using endoscopy and pathology databases, named the Western Honduras Gastric Cancer Prevention Initiative (WHGCI).^16^ This served as the foundation for the multi-institutional collaboration of the Western Honduras PBCR (Copán-PBCR): principal public hospital and clinic system (Hospital de Occidente, HdO), local academic institutions (Universidad Nacional Autónoma de Honduras), the Honduran Ministry of Health, and international universities (University of Alabama at Birmingham, Vanderbilt University, and Universidad del Valle, Cali, Colombia). This article describes the implementation of the Copán-PBCR and provides estimates of the initial 5 years.

## METHODS

Western Honduras comprises three departments (states): Copán, Ocotepeque, and Lempira. The Copán-PBCR is specific to the state of Copán. The department of Copán represents 5% of Honduras population and 44% of the western population (382,722; 189,172 females and 193,550 males).^25^ The health infrastructure consists of one principal regional hospital (WRH) and affiliated clinics, which provides surgical oncology services, digestive endoscopy, cervical and breast cancer screening, diagnostic imaging, and pathology services. Clinical oncology, chemotherapy, and radiotherapy services are currently unavailable in the west.

Incident cancer cases were identified from three main sources: (1) the hospital Cancer Prevention Program, (2) monthly notifications from the Honduran Ministry of Health, and (3) cases reported by all public and private institutions in Western Honduras, which offer cancer diagnostic services (rural clinics and pathology laboratories). All incident cases were recorded from January 1, 2013, to December 31, 2017. Mortality data are not available at the regional nor national levels (eg, National Population Registry [RNP]), as death certificates in Honduras are incomplete and of low quality. Residents of the state of Copán were included with address verification (RNP) and records from the diagnostic centers. Protocols were in place to maintain confidentiality and protect personal health information, per international standards.

The cancer case definition was any incident invasive malignant tumor from any anatomical location confirmed regardless of treatment status. The diagnosis was either microscopic (fluid cytology, peripheral blood and bone marrow, histology of primary tumors, or autopsy) or nonmicroscopic (clinical, surgical, or imaging). The following cancers were included: single or multiple primary malignant tumors, all tumors of the central nervous system, in situ breast and cervical cancer, melanoma and nonmelanoma skin cancer (NMSC). Benign tumors with uncertain behavior and malignant tumors of metastatic sites were excluded. The basis of the diagnosis and all variables included in IARC's standard for cancer registries were precoded. Tumor or cancer site, morphology, behavior, degree of differentiation, and incidence dates were recoded according to the descriptions supplied by the entities.^26^

Quality reviews were performed to verify codes for demographic, topographic, and morphologic variables (eg, age-birth-incidence date, sex-site, sex-histology, age-site, age-histology, site-histology, and basis of diagnosis-histology). Duplicate records were eliminated on the basis of the National Identification Number and medical record numbers. Diagnoses were summarized according to the International Classification of Diseases Volume 10 (ICD-10)^27^ and International Classification of Diseases-Oncology-third edition (ICD-O-3) classification.^20^

The crude, age-specific, and world population age-standardized rates (ASRs) were calculated and expressed per 100,000 population, using the Segi-Doll world standard methodology, according to the Cancer Incidence in five continents (CI5) recommendations.^10^ Estimates are presented as three-character ICD-10 codes using the format recommended by the CI5 X grouping for ICD codes.^28,29^ Cancer incidence rates are presented with and without NMSC(C44). Age-specific incidence rates were calculated for both sexes, male and female, on the basis of national census. The most recent national census was conducted in 2013 by the Honduras National Statistics Institute (INE), with official estimates for subsequent years (eg, 2014-2017). The census represents the geographical strata of the local population with a limited censal omission of 10.3%.^25^

Innovations included the use of an alternate data management platform and leverage of Health Ministry personnel resources. We designed the PBCR database using the web-based Research Electronic Data Capture (REDCap) platform, which required minimal training. The online database facilitated collaboration with real-time access to deidentified registration data for quality checks and for feedback. In the pilot phase, we noted improved efficiency and quality measures when compared with local use of CanReg5 (data not shown). In the interest of capacity building, we piloted the inclusion of recently trained physicians who were in their required year of national service (social service) in need areas with a focus on the rural areas. Data analysis was performed using STATA 13 (Stata Corp, College Station, TX). The study protocol was approved by the IRBs of the Honduras Western Regional Hospital and Vanderbilt University.

## RESULTS

The Copán-PBCR registered 1,812 new cancer diagnosis in the study period. The annual crude incidence rates for the 5-year period in the Western Honduras for all sites excluding NMSC (C44) were 112.2, 69.8, and 154.6 per 100,000 habitants overall, males, and females, respectively. The ASRs excluding NMSC were 84.2, 49.6, and 118.9 per 100,000 habitants overall, males, and females, respectively (Table 1). The crude cancer rates and ASRs are presented in Table 2 (males) and Table 3 (females) organized by the most common ICD-10 sites. Detailed estimates are also provided for all sites (Data Supplement). There were no significant differences in annual rates among the three most common cancers (stomach, cervix, and breast) during the 5-year period. The ASRs in the border municipalities may be underestimated as patients may seek care in Guatemala or El Salvador.

**TABLE 1:**
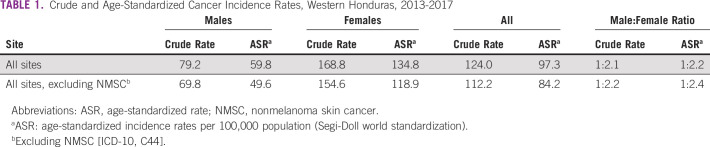
Crude and Age-Standardized Cancer Incidence Rates, Western Honduras, 2013-2017

**TABLE 2:**
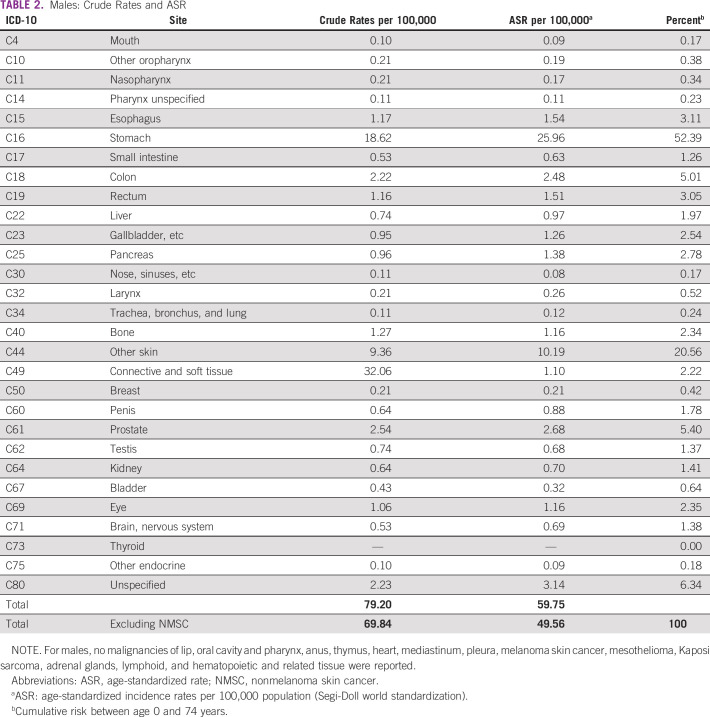
Males: Crude Rates and ASR

**TABLE 3:**
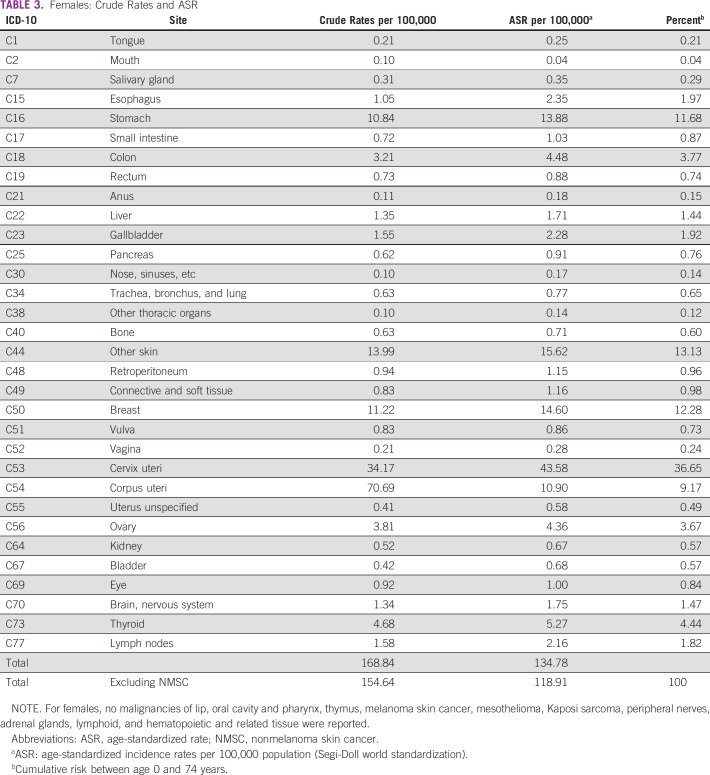
Females: Crude Rates and ASR

**FIG 1:**
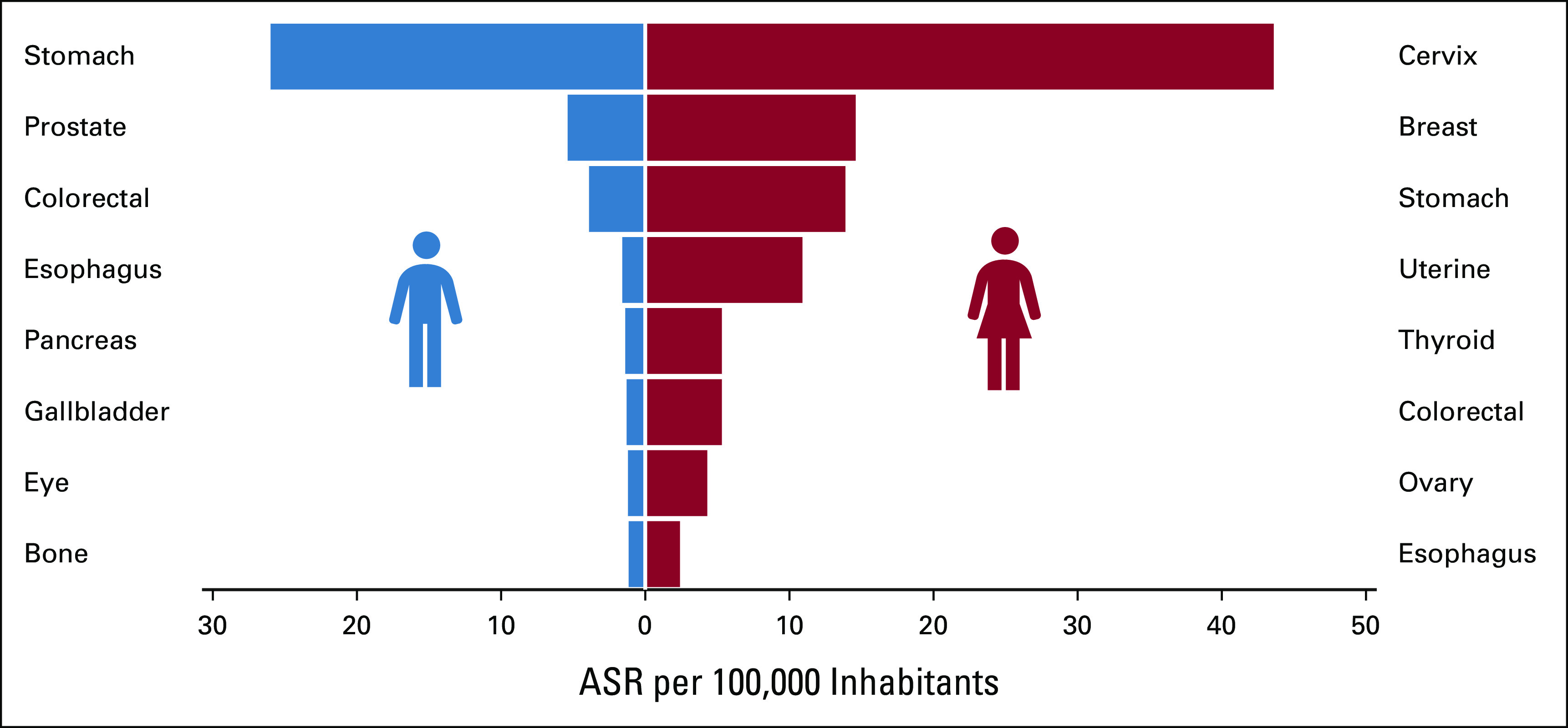
Most common cancer sites, male and female, Western Honduras, 2013-2017. ASR: age-standardized incidence rates per 100,000 population (Segi-Doll world standardization), excluding nonmelanoma skin cancer.

The incidence of the most frequent cancers is summarized in Figure 1. The leading cancers in males were stomach cancer (ASR = 26.0), colorectal cancer (ASR = 5.1), and prostate (ASR = 2.7), which represented 62.8% of cancer in men. In females, cervix uteri (ASR = 43.6), breast (ASR = 14.7), and stomach (ASR = 13.9) were the most common, accounting for nearly 60%. The estimated lifetime risk (age 0-74 years) of developing stomach cancer was 3.4% in males and 1.4% in females. In females, the lifetime risk of developing cervix uteri cancer was 3.1%. Two hundred ten cancer records (11.6%) had missing or incomplete data. In 1,597 (82.6%) cancer reports, the diagnosis was verified with histopathology reports. Only 54 (0.03%) cases were based upon the death certificate.

## DISCUSSION

We present the initial cancer incidence estimates from the Copán-PBCR over a 5-year period, in an effort to advance cancer control in Honduras and the CA-4 region. The ASRs for both sexes were 124 and 112.2 per 100,000 including and excluding NMSC (C44), respectively. The gender difference (1:2.4 male-female ratio) was driven by cervical cancer. The highest ASRs were cervical cancer (43.6/100,000) and breast cancer (14.6/100,000) among females and stomach cancer in males (26/100,000). The rates in the major cancers did not significantly vary during the 5-year period. There was modest geographic variation, with lower rates observed in more remote municipalities, suggesting that access to diagnostic services may play an important role. Innovations included the use of the REDCap software platform with online and offline secure data entry and the inclusion of recently trained physicians in their required year of rural national service for network support.

Cancer incidence in the CA-4 is modest compared with Europe, North America, and other high-income nations,^13^ because of the young demographic pyramid and high mortality from communicable diseases. The cancer profile in rural Western Honduras is similar to the profile seen in Latin American middle-income countries (eg, Colombia) 30-50 years ago.^30^ Rural Honduras is now experiencing lifestyle westernization with dietary changes, urbanization, sedentarism, socioeconomic improvement, delayed childbearing, and decreased parity, which forebode changes in its cancer profile.^27^ Our data suggest the emergence of the double burden of cancer: high incidence of infectious-driven cancers (cervical and stomach) with increasing rates of cancer associated with modernization, obesity, and aging (breast and colorectal).^26,31,32^

Our study confirms the ongoing challenge of cervical cancer in Honduras, now compounded by breast cancer risk. The ASR in rural Honduras is nearly double than that estimated by GLOBOCAN 2018 (45.58, 29.7). The incidence is likely multifactorial and caused by the high prevalence of HPV, high-risk HPV types, and household exposures to carcinogens (wood stoves).^33,34^ The observed breast cancer ASR was lower than that reported in the GLOBOCAN 2018 estimates (14.6, 31.1). Lower reported breast cancer rates may be explained by the lack of infrastructure and resistance toward screening because of cultural beliefs and health literacy. Women living in the CA-4 suffer from educational and economic exclusion.

Stomach cancer had the highest incidence for males and was second for females (26.0 and 13.9 per 100,000, respectively), higher than that reported by GLOBOCAN 2018 (12.7, 9.7). These estimates are consistent with previous studies in Honduras with the incidence of 30.8 and 13.9 in males and females.^16^ The high incidence of gastric cancer has been attributed to endemic *Helicobacter pylori* infection with virulent strains (eg, cagA), germline genetic influences, and environmental exposures (eg, wood stoves).^35-37^

Colorectal and prostate cancers were the second and third most frequent cancer in males (5.1 and 2.7 per 100,000, respectively), at rates lower than that estimated by GLOBOCAN 2018.^13^ This may be attributed to lack of screening strategies, access to care, and under-reporting. There are no urology providers in the area, and patients must travel outside of Copán for care.^38^ Although lower than North America, the colon cancer estimates are similar to those reported in Latin America over the past decade.^39,40^

There is a substantial gap in IARC CI5 coverage between low- and middle-HDI countries (5%-10% coverage in South America and no coverage in the CA-4) and high-HDI countries (35% coverage in United States).^27^ In South and Central America, only 11 countries submitted cancer data to the Cancer in Five Continents Report. Specifically, for Central America, only Costa Rica provided data in the past 5 years.^10,20^

The design and implementation of the PBCR faced several challenges with lessons learned: (1) Using CanReg5 for data collection in public and private institutions and pathology laboratories was difficult to coordinate. The REDCap platform permitted straightforward training of personnel and efficient data entry available online and offline. (2) The Honduras National Population (Census) Registry has under-registration of residents in Western Honduras, and cause of death is not recorded. This was overcome by active follow-up of case mortality. (3) Cancer registration in Honduras is not mandatory. This was addressed by working with the Copán Ministry of Health to approve an ordinance for all health institutions that diagnose or treat cancer in the area to submit monthly cancer reports. (4) Institutional support from the Ministry of Health was limited. In addition to the academic collaborations, local resources were leveraged. One example is the inclusion of recently trained physicians for reporting network support, who were in their required year of rural national service, which also served as a capacity building and education initiative for these trainees. These lessons learned and innovation in Honduras parallel other LMIC experiences and suggest that the Copán-PBCR can be strengthened^41,42^ and is critical in countries with few physicians per capita (only 0.3 per 1,000 in Honduras).^43^

The success of the first five years of the Copán-PBCR is the result of collaborative partnerships between local and international institutions, which are expected to continue. Collaboration between academic institutions in the United States, Colombia, and Honduras was strengthened in the 2000s with the development of the National Cancer Institute–funded gastric cancer epidemiology program (WHGCI), which provided training and infrastructure to physicians, researchers, and data collectors and served as a cornerstone of the Copán PRBC.^44^

In 2014, the IARC Global Initiative for Cancer Registry Development (GIRC) founded a hub for Latin America. The Argentina-based hub seeks to develop cancer registries across Latin America. Since 2015, the GIRC has conducted visits to Central America, offering training in Honduras, El Salvador, and Guatemala. We foresee a stronger collaboration between the GIRC-Latin American Hub and the Copán-PBCR. This collaboration should improve data quality and coverage and guide future planning. Although GIRC trainers have been based in South America, CA-4–based trainers could be beneficial for the region.^45^ The cancer data generated by the Copán-PBCR could be integrated to the Network of National Cancer Institutes of Latin America (RINC-ALC), which has a focus on cervical cancer.

Global projections foresee a marked rise of cancer incidence in transition economies (four fifths of the cancer burden falling among these regions by 2025), including Central America.^12,32^ Improved living conditions may decrease the burden of some infectious and environment-driven cancers; however, policies that address common cancer risk factors (eg, obesity, tobacco, and alcohol) are a critical part of cancer prevention, given the emerging double cancer burden in LMICs.^46^

The geographic coverage of the Copán-PBCR is modest. Western Honduras (Copán, Lempira, Santa Barbara, and Ocotepeque) accounts for approximately 1.5 rural million habitants (16% of the country's population). Expanded geographic coverage to include the neighboring states is feasible, but sustained support from the central government, academic institutions, or international agencies is needed.

Copán is a rural area, and inferences to the urban areas in the country or the CA-4 should be cautious.

The Copán-PBCR has certain limitations related to completeness. Studies suggest that combining active and passive registration provides a balance of adequate registration, with lower costs and limited bias, and is expected to continue in Copán.^47,48^ The unknown cause of death or not attributed to cancer may be due to limited diagnostics and low-quality national death registration. Some patients seek care in the Honduras urban centers or in the neighboring nations (Guatemala and El Salvador). Permanent international migration constituted 1.7% of the region's population in the 2013 census and may increase in 2021.^25^ Of note, there are limitations in the diagnosis of certain malignancies in Western Honduras. Endoscopy services and gynecologic consultations are readily available. Other cancers like intrathoracic malignancies (eg, lung cancer), intracranial tumors, urologic tumors, and hematologic neoplasia remain difficult to confirm locally, and the active ascertainment is helpful.

In conclusion, the Copán-PBCR represents a successful model to establish cancer registration in rural LMICs, provides the first population-based cancer estimates in the CA-4 region, and contributes to the GICR and IARC initiatives. We used a web-based approach with the REDCap platform that facilitated data collection and enhanced quality checks. Infection-associated cancers such as gastric and cervical cancer have high incidence in this region, making them priority targets for cancer control initiatives. Emerging cancers, including breast and colorectal, portend the future double cancer burden common to transitioning economies in Latin America.
